# Divergent Effects of Human Cytomegalovirus and Herpes Simplex Virus-1 on Cellular Metabolism

**DOI:** 10.1371/journal.ppat.1002124

**Published:** 2011-07-14

**Authors:** Livia Vastag, Emre Koyuncu, Sarah L. Grady, Thomas E. Shenk, Joshua D. Rabinowitz

**Affiliations:** 1 Department of Chemistry, Princeton University, Princeton, New Jersey, United States of America; 2 Department of Molecular Biology, Princeton University, Princeton, New Jersey, United States of America; University of Washington, United States of America

## Abstract

Viruses rely on the metabolic network of the host cell to provide energy and macromolecular precursors to fuel viral replication. Here we used mass spectrometry to examine the impact of two related herpesviruses, human cytomegalovirus (HCMV) and herpes simplex virus type-1 (HSV-1), on the metabolism of fibroblast and epithelial host cells. Each virus triggered strong metabolic changes that were conserved across different host cell types. The metabolic effects of the two viruses were, however, largely distinct. HCMV but not HSV-1 increased glycolytic flux. HCMV profoundly increased TCA compound levels and flow of two carbon units required for TCA cycle turning and fatty acid synthesis. HSV-1 increased anapleurotic influx to the TCA cycle through pyruvate carboxylase, feeding pyrimidine biosynthesis. Thus, these two related herpesviruses drive diverse host cells to execute distinct, virus-specific metabolic programs. Current drugs target nucleotide metabolism for treatment of both viruses. Although our results confirm that this is a robust target for HSV-1, therapeutic interventions at other points in metabolism might prove more effective for treatment of HCMV.

## Author Summary

Enveloped viruses draw on cellular machinery and materials to generate copies of their genome, structural proteins, and membrane. These biosynthetic processes use the host metabolic network to provide energy and small-molecule precursors. We have investigated how two important enveloped viruses, human cytomegalovirus and herpes simplex virus-1, alter host metabolism to provide materials for viral replication. We show that rather than passively relying on basal host cell metabolic activity, both viruses actively redirect host cell metabolism, implementing divergent metabolic programs that are robust to host cell type and virus strain. Human cytomegalovirus enhances lipid biosynthesis, while herpes simplex-1 gears central carbon metabolism toward the synthesis of pyrimidine nucleotides. Consistent with these changes, human cytomegalovirus is more sensitive to inhibition of fatty acid synthesis and herpes simplex virus-1 to inhibition of central metabolic reactions leading towards pyrimidine synthesis. As these two closely related viruses have divergent metabolic strategies, and since the metabolic perturbations point to potential drug targets, an important priority is defining the metabolic programs of other viruses.

## Introduction

Herpesviruses are large, enveloped, double-stranded DNA viruses, capable of both lytic infection and life-long latency in mammalian hosts [1]. They are major causes of human disease. A majority of adults are infected with herpes simplex virus 1 (HSV-1) and/or human cytomegalovirus (HCMV). An alpha-herpesvirus, HSV-1 infects a wide range of organisms and cells types, causing symptoms ranging from cold sores to encephalitis. The prototypical beta-herpesvirus, HCMV, selectively infects non-transformed human cells. Although frequently asymptomatic, HCMV causes severe disease in neonates and immunocompromised adults. All herpesviruses encode metabolic enzymes in their genomes, primarily ones involved in nucleotide metabolism. The HSV-1 genome encodes a viral thymidine kinase, ribonucleotide reductase, dUTPase and uracil DNA glycosylase, while HCMV encodes a functional form of uracil DNA glycosylase [2]. Like all viruses, however, they rely primarily on the metabolic capabilities of their cellular hosts for replication. Specifically, the host provides the energy, amino acids and lipids, as well as most nucleotides, required by the virus.

Improved technologies for measuring both enzymes and metabolites is enabling for the first time in-depth analysis of virus-host cell metabolic interactions. Liquid chromatography coupled to mass spectrometry (LC-MS) facilitates direct measurement of a large number of cellular metabolites [3], [4]. Combined with isotope tracers, metabolic flows (fluxes) can also be determined. These new tools have revealed that, rather than passively relying on basal host cell metabolic activity, many viruses actively redirect host cell metabolism [5], [6], [7]. For example, hepatitis C virus up-regulates host cell glycolysis and modulates concentrations of specific lipids [8]. Similarly, hepatitis B virus replication perturbs cholesterol metabolism by inducing increased 7-dehydrocholesterol levels [9].

Among herpes viruses, the metabolic effects of HCMV have been the most extensively studied. Infection of a human fibroblast cell line with HCMV leads to two-fold increases in glycolytic activity and nucleotide synthesis, as well as yet greater increases in citric acid cycle flux and lipid biosynthesis [10]. Consistent with HCMV's reliance on the metabolic fluxes that it induces, inhibition of the committed step of fatty acid synthesis and elongation, acetyl-CoA carboxylase, blocks HCMV replication [10]. The virus also induces an increased dependence on glutamine that serves to drive the TCA cycle [10], [11]. These metabolic changes are only partially accounted for by increased levels of enzyme transcripts, indicating the involvement of multiple regulatory mechanisms [6].

A limitation of studies of virus-host metabolic interactions to date is that they have focused on single virus-host cell pairs. Moreover, they have often employed transformed host cells that differ markedly from the cells usually infected *in vivo*. This has precluded understanding whether the observed metabolic effects of viruses are relevant in their natural host cells, preserved across host cell types, and conserved within families of related viruses. To address these issues, here we compare and contrast the metabolic effects of HCMV and HSV-1, across both fibroblast and epithelial host cells. Specifically, we studied the laboratory-adapted AD-169 strain of HCMV, which is restricted to growth in fibroblasts, and whose metabolic effects have been previously studied [6], [10]. In addition, we examined the epitheliotropic clinical isolate strain TB40/E, which grows in many cell types, to study the infection of epithelial cells [12]. For HSV-1 infections, we chose the highly-passaged, non-neuroinvasive KOS 1.1 strain and a prototypical neuroinvasive strain, the F strain [13], [14]. Both primary human foreskin fibroblasts and the MRC5 fibroblast cell line were analyzed after infection by both viruses. HSV-1 infection was also studied in the Vero African green monkey renal epithelial cell line which is traditionally used for its growth. Given HCMV's propensity to cause retinitis [15], it was studied in the ARPE-19 retinal pigment epithelial cell line.

Using LC-MS to probe core metabolite concentrations and fluxes, we find that HCMV and HSV-1 both trigger major metabolic changes in their cellular hosts, and that these changes are similar across different host cell types and for different strains of the same virus. In contrast, the effects of HCMV and HSV-1 diverge markedly. HCMV most greatly impacts pathways generating substrates for lipid metabolism, whereas HSV-1 most greatly impacts deoxypyrimidine metabolism.

## Results

### HCMV and HSV-1 trigger distinct metabolome changes

We examined the metabolic changes triggered by infection of fibroblast and epithelial host cells with HCMV and HSV-1. Fibroblasts (HFF and MRC5) were held at confluence for 3–5 days then serum-starved for 24 hours prior to infection, while epithelial cells (ARPE19 and Vero) were infected at 80–90% confluence and maintained in the presence of dialyzed serum at all times. As a consequence the fibroblast host cells were growth arrested at the time of infection, while the epithelial cells continued to replicate after mock inoculation [16]. Consistent with their different growth states, there were substantial differences in the metabolome of the host cells prior to infection, with compounds directly involved in proliferative processes such as carbamoyl-aspartate (pyrimidine biosynthesis), dTTP (DNA synthesis), and S-methyl-5′-thioadenosine (polyamine synthesis) markedly higher in the growing epithelial cells than the quiescent fibroblasts (Figure S1). Other compounds, such as those involved in mitochondrial fatty acid oxidation (carnitine and acetyl-carnitine), were higher in growth arrested fibroblasts compared to growing epithelial cells.

Infections were performed at a multiplicity of 3 pfu/cell to ensure near complete exposure of the cell population. Cultures infected with one of the virus strains, or treated with a virus-free mock inoculum, were grown in parallel and sampled at regular time intervals from the beginning of infection until peak virus yields were achieved. Medium was changed every 24 h to ensure a consistent nutrient supply to the cells; lack of media changes in earlier work [6] resulted in some different metabolite patterns from those observed here. In particular, we find that citrate and malate levels increase >10-fold during HCMV infection, compared to the 2-fold change seen in previously published work [6]. Maximum virus output was reached at around 24 h post infection (hpi) in HSV-1 infected cells, and around 96 hpi HCMV infected cells (Figure S2).

**Figure 1:**
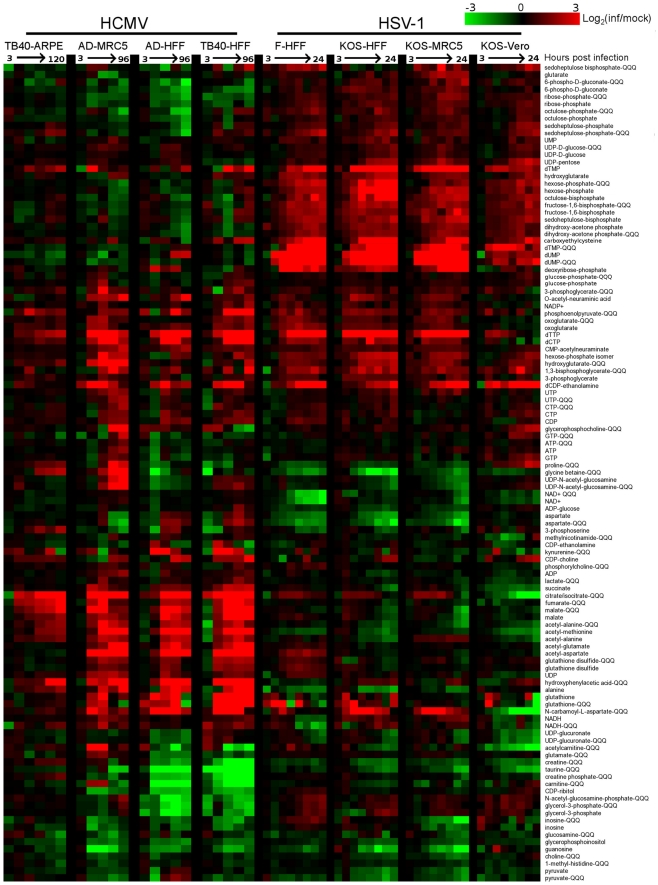
Divergent metabolic profiles of HCMV- and HSV-1-infected cells. Metabolite levels during the course of HCMV and HSV-1 infection, normalized by packed cell volume and expressed relative to levels measured in the equivalent mock-treated host cells. Ratios are log transformed and plotted on a color scale. Rows correspond to metabolites measured either by LC-high resolution MS or LC-triple quadrupole MS/MS (those measured by triple quadruople are marked “QQQ”). Columns correspond to hours post infection for each of the eight infection time courses. The host cells and virus strains used in each time course are indicated. HCMV (strains TB40/E and AD169) and HSV-1 (strains KOS and F) were used to infect growing epithelial (ARPE19 and Vero) and growth arrested fibroblasts (HFF and MRC5). During HCMV infection samples were taken at 3, 24, 48, 72, 96 hpi, and also at 120 hpi during the infection of ARPE19 cells with the TB40/E strain. During HSV infection samples were collected at 3, 6, 9, 12, 15, 18, 21, and 24 hpi. Values are averages of duplicate independent biological experiments. To view the same figure in blue-yellow color scale, see Figure S1.

Over 80 metabolites were identified and detected in all experiments. Relative concentrations of these species, between infected and mock-infected cells, are shown in Figure 1 (for blue/yellow version of the heat map, see Figure S3; for source data, see Table S1). A third of the compounds were measured by both high resolution mass spectrometry (orbitrap) and triple quadrupole mass spectrometry (QQQ). The profile of any single metabolite detected in multiple LC-MS methods was found to be similar, as indicated by co-clustering of the associated data in almost all cases (Figure 1).

Both viruses triggered >4-fold changes in the levels of roughly half of the metabolites assayed. Among the metabolites changing markedly, those increasing outnumbered those decreasing roughly two-to-one. Although the magnitude of the changes in compound levels depended on the host cell, typically being smaller in the growing epithelial cells, the majority of the trends were host cell invariant. This is remarkable given the differing initial growth and metabolic states of the host cells, and it indicates a robust ability of the viruses to re-program metabolism.

Extracting major trends from the dataset by singular value decomposition [17] resulted in two characteristic vectors that accounted for >10% of the information in the dataset (Figure S4A). These vectors represent prototypical metabolite response patterns. The first vector accounts for 16% of the variation in metabolite levels over the time courses. In this vector, the signal as a function of time shows a similar trend in each of the time courses, thus representing a generic metabolite concentration response to herpesvirus infection (Figure S4B). The smaller signal in the first and last columns corresponding to the infections of epithelial cells reflects the smaller fold-changes in metabolite levels induced by viral infection in the growing epithelial cells compared to growth arrested fibroblasts. The strongest contributor to the generic infection response is dTTP, whose upregulation is consistent with the shared need of both viruses to replicate their DNA. The second vector, accounting for 12% of the variation in the dataset, represents a virus-specific response with opposing patterns for the HCMV and HSV-1 infection time courses (Figure S4B). Key contributors to this virus-specific response include TCA cycle intermediates, consistent with their rise during HCMV but not HSV-1 infection, and the nucleotides dUMP and dTMP, consistent with their rise during HSV-1 but not HCMV infection.

The third most significant vector, which accounts for 6% of the information in the dataset, represents a metabolic response characteristic of Vero cells (Figure S4B). While most of the changes proved to be host cell-independent, the third vector draws attention to the impact of different host cell types on the metabolic effects of viruses. The strongest contributors to this vector are citrate/isocitrate and N-carbamoyl-L-aspartate, due to their depletion in infected Vero cells in contrast to their accumulation in all other cell types. The remaining characteristic vectors account for the other 66% of the information. This large amount of residual information reflects a myriad of metabolite, virus, and cell-type specific dynamics. For example, proline and glycine-betaine showed cell type-specific upward or downward trends. Other metabolites, such as dTTP, showed different dynamic response patterns across different host cell types.

In all cell types tested, HCMV infection induced phosphoenolpyruvate, deoxypyrimidine triphosphates, CDP-choline, and acetylated amino acids, as well as a striking and coordinated increase in citrate, malate and other TCA cycle intermediates (Figure 1). Depleted compounds included glycerophosphoinositol, taurine, and a number of pentose phosphate pathway metabolites. On the other hand, HSV-1 triggered increased levels of pentose phosphate pathway intermediates, as well as glycolytic intermediates, and deoxypyrimidines (Figure 1). Notably depleted compounds included glycine betaine, taurine, creatine, and NAD+. The conserved decrease in the osmolyte, taurine, in both HCMV and HSV-1 likely reflects a host cell response to virus-induced increases in cell volume [18]. Glycolysis, the citric acid cycle, and pyrimidine biosynthesis are discussed in greater detail below.

### Glycolytic flux during HCMV and HSV-1 infection

**Figure 2:**
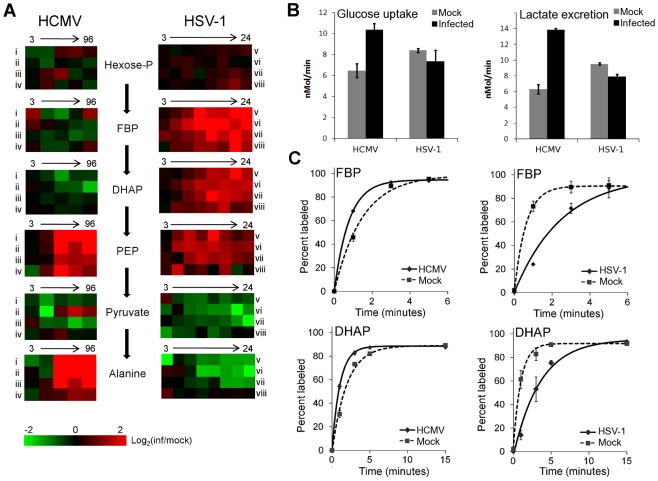
Perturbation of glycolysis by HCMV and HSV-1. (**A**) Plots of individual metabolite abundance during HCMV and HSV-1 infection. Data are the same as presented in Figure 1. Metabolite concentrations are expressed relative to equivalent mock-treated cells. Rows correspond to infection time courses of the following virus strains and cell types: (i) TB40-HFF, (ii) AD169-HFF, (iii) AD169-MRC5, (iv) TB40-ARPE19, (v) F-HFF, (vi) KOS-HFF, (vii) KOS-MRC5, (viii) KOS-Vero. Columns correspond to time points: 3, 24, 48, 72, 96 hpi for HCMV and 3, 6, 9, 12, 15, 18, 21, 24 hpi for HSV-1 (Hexose-P: glucose-6-phosphate and its isomers; FBP: fructose-1,6-bisphosphate; DHAP: dihydroxy acetone-phosphate; PEP: phosphoenolpyruvate). (**B**) Measurement of glucose uptake and lactate excretion rates in HCMV-AD169 or HSV-KOS infected, as well as mock-treated, human foreskin fibroblasts (mean ±2 s.e.; n = 3). (**C**) Buildup of the labeled fraction of the FBP and DHAP pools after switching cells to uniformly ^13^C-labeled glucose medium at 12 hpi during HSV-KOS or 48 hpi during HCMV-AD169 infection or equivalent virus-free treatment of HFF cells. Symbols indicate experimental data points ±2 s.e.; n = 2; lines indicate exponential fit.

Glycolysis and the TCA cycle form the backbone of central carbon metabolism in mammalian cells. Through these two pathways glucose is either oxidized to produce energy in the form of NADH and ATP, or converted to precursors of amino acids, lipids and nucleotides. The levels of glycolytic intermediates are altered in a strikingly different manner during HCMV and HSV-1 infections (Figure 2A). The concentrations of metabolites in lower glycolysis increase during HCMV infection, while levels of upper glycolytic intermediates drop. Conversely, in response to HSV-1 infection the opposite occurs. While these concentration measurements are informative, it is not possible to deduce whether changes in influx, efflux or a combination of both are responsible for the perturbations of the metabolite levels. Neither the turnover rate of a metabolic intermediate, nor the material flow through a pathway, can be predicted based on metabolite pool sizes alone. To understand how material flow, i.e., flux, is altered, further assays must be employed.

In cultured mammalian cells, the enzyme-catalyzed reactions of glycolysis convert the bulk of glucose imported from the extracellular environment to lactate, which gets excreted. Thus, changes in the rate of material flow through glycolysis can be approximated by measuring the rate of glucose consumption and lactate production. We determined the glucose uptake and lactate excretion rates in infected and mock treated HFFs by directly measuring the amount of glucose and lactate in the extracellular medium over time (Figure 2B). HCMV increased the uptake of glucose (p = 0.02) and the excretion of lactate (p = 0.0006), in agreement with previously published results on HCMV-infected fibroblasts [10], [19], [20] (Figure 2B). On the contrary, in HSV-1 infected cells the glucose uptake (p = 0.21) and lactate excretion (p = 0.002) rates decreased to a modest extent.

In addition to glucose from the medium, glycolysis can also be fueled by glucose acquired from the breakdown of stored glycogen. Moreover, decreased lactate production can reflect increased glycolytic efflux to the TCA cycle, rather than decreased glycolytic flux. To confirm our conclusions based on the glucose and lactate measurements, we also measured the rate of incorporation of isotope-labeled nutrients into downstream metabolites. Following a switch to labeled media, metabolite pools become progressively more labeled, with the unlabeled fraction exhibiting an exponential-type decay. Flux through a metabolite is the product of the rate of this decay and the total pool size of the metabolite [21]. To reliably estimate this decay rate, it is important to obtain samples at early time points where the fractional labeling is changing rapidly. Because label from glucose gets incorporated very quickly into glycolytic intermediates, measurements at later time points are likely to reflect steady-state labeling fractions, not labeling rates per se. At steady state, the amount of labeled metabolite reflects the total metabolite pool size and the fraction of its production from the labeled substrate, but not the rate of labeling.

To characterize glycolytic flux, we switched HCMV and HSV-1 infected cells, as well as their mock-treated counterparts, to ^13^C-labeled glucose containing media, and used LC-MS to monitor the labeled forms of downstream metabolites over time. HCMV infection increased the fractional labeling rate of glycolytic intermediates fructose-1,6-bisphosphate and dihydroxyacetone phosphate, while HSV-1 decreased it (Figure 2C). The decrease in the rate of HSV-1 labeling was complemented by a corresponding increase in metabolite concentration. Thus, we can conclude that HCMV significantly increases flux through glycolysis and HSV-1 does not.

Interestingly, in HSV-1-infected cells the metabolites upstream of phosphoenolpyruvate build up, while the ones downstream drop (Figure 2A). This suggests a bottleneck in glycolytic efflux at the step catalyzed by pyruvate kinase, the enzyme that converts phosphoenolpyruvate and ADP to pyruvate and ATP. The buildup of glycolytic metabolites upstream of pyruvate is accompanied by increased levels of pentose phosphate pathway intermediates, thus increasing the availability of ribose-phosphate for the synthesis of nucleotides. During hepatitis C infection the levels of most glycolytic enzymes were shown to be elevated, with the notable exception of pyruvate kinase [8]. Such changes in enzyme levels may lead to a similar metabolic outcome as observed in HSV-1 infected cells. However, as the activity of glycolytic flow is under tight allosteric control [22], direct metabolic analysis of hepatitis C is warranted to confirm this.

### TCA cycle influx during HCMV and HSV-1 infection

**Figure 3:**
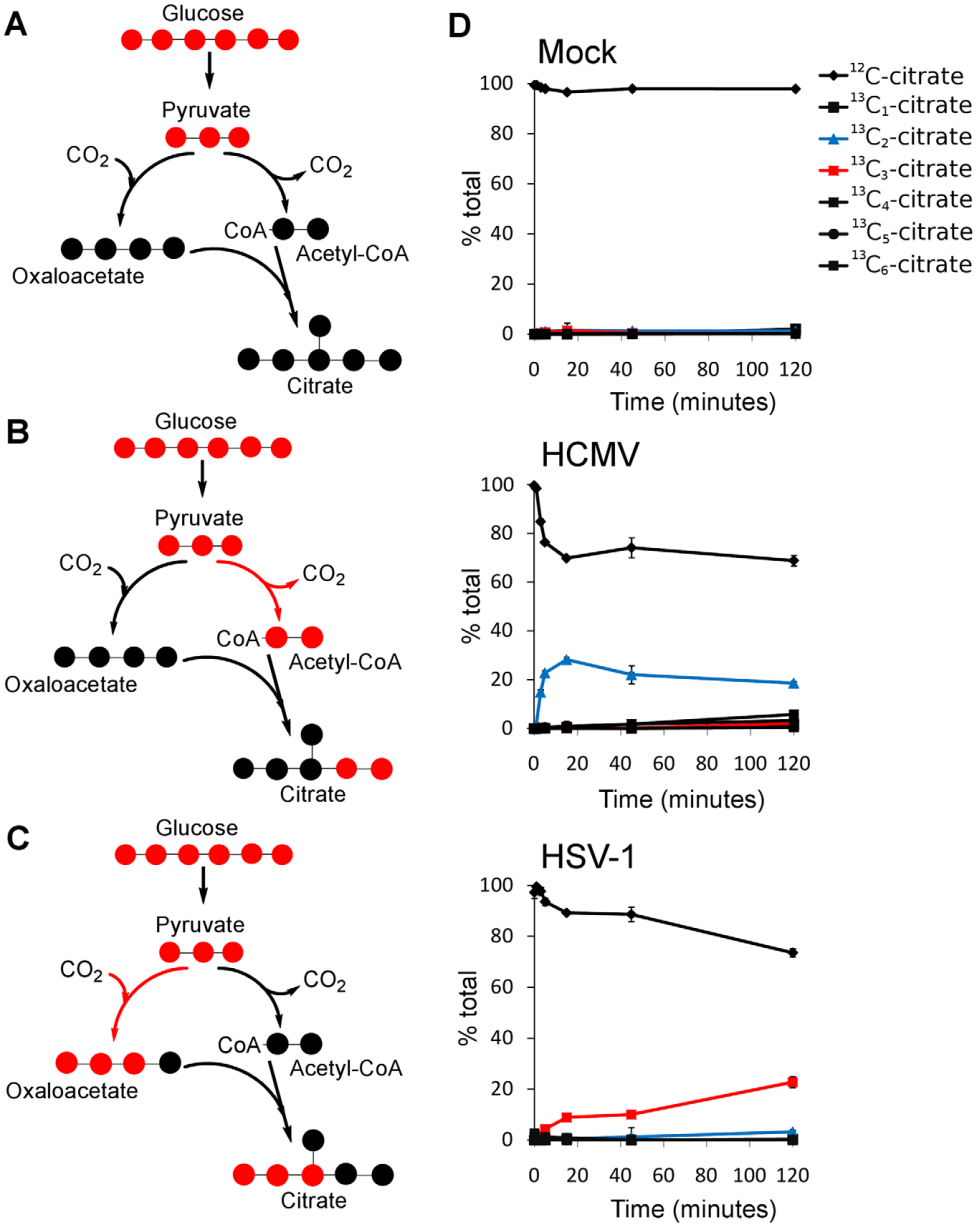
Virus-specific up-regulation of glucose influx to the TCA cycle. The left column of schematic show carbon labeling from glucose to the TCA cycle via pyruvate carboxylase and pyruvate dehydrogenase. Red dots denote ^13^C atoms originating from uniformly ^13^C-labeled glucose. (**A**) Labeling patterns when neither pyruvate carboxylase nor pyruvate dehydrogenase are active. (**B**) Labeling pattern when carbon influx to the TCA cycle from glucose is via pyruvate dehydrogenase. (**C**) Labeling pattern when carbon influx to the TCA cycle from glucose happens via pyruvate carboxylase. (**D**) Levels of various labeled forms of citrate expressed as percent of the total citrate pool upon switching HCMV-AD169, HSV-KOS or mock-treated HFF cells to uniformly ^13^C-labeled glucose medium. HCMV infected cells were switched at 48 hpi, HSV-1-infected cells at 12 hpi. The x-axis indicates time after switching to labeled medium (mean ±1 s.d.; n = 2).

The metabolites of the TCA cycle showed a particularly interesting difference in labeling patterns when HCMV- and HSV-1-infected fibroblasts were supplied with uniformly labeled ^13^C- glucose. In the uninfected, growth arrested fibroblasts, citrate was only minimally labeled over a 2 h time period (Figure 3D, top panel). On the other hand, HCMV-infected fibroblasts produced a significant amount of citrate with two labeled carbon atoms (^13^C_2_-citrate) (Figure 3D, center panel), while their HSV-1-infected counterparts generated citrate with three labeled carbons (^13^C_3_-citrate) (Figure 3D, bottom panel). These two forms of citrate are produced by different pathways, which are selectively up-regulated in a virus-specific manner.

Labeled carbon atoms derived from ^13^C-glucose can enter the TCA cycle via two routes (Figure 3A). In one, pyruvate dehydrogenase and citrate synthase incorporate two carbons from glucose into citrate via acetyl-CoA (Figure 3B). The labeling pattern of citrate during HCMV infection indicates increased influx of glycolytic carbon to the TCA cycle via this route (Figure 3D). This pathway indicates a catalytic use of the TCA cycle, with the two-carbon units originating from glycolysis either oxidized to produce energy by complete turning of the TCA cycle, or diverted from the mitochondria to the cytosol through the citrate shuttle, where the acetyl group is freed for fatty acid synthesis and/or elongation. Global flux analysis on HCMV infection showed that both of these uses of glycolytic carbon are up-regulated by HCMV in MRC5 cells [10]. Our results indicate that HCMV infection of HFFs leads to the same up-regulation.

Carbon from glycolysis can also enter the TCA cycle via pyruvate carboxylase, which converts pyruvate to oxaloacetate (Figure 3C). All three labeled carbons in pyruvate are retained in oxaloacetate, which is converted to ^13^C_3_-citrate, malate, or aspartate. The labeled forms of TCA cycle intermediates observed in HSV-1-infected cells indicate an up-regulation of carbon influx via pyruvate carboxylase as reflected by the labeling of citrate (Figure 3D, right panel) and malate (Figure S5) when cells are supplied with ^13^C_6_-glucose. Furthermore, no citrate is detected with two or five labeled carbons in these cells. Thus, unlike in HCMV-infected cells, the use of glucose to drive the citrate shuttle and ensuing fatty acid synthesis is minimal during HSV-1 infection. The previous metabolic analysis of HCMV infection led to the recognition of a potential new drug target by showing that *de novo* fatty acid biosynthesis is essential for HCMV replication [10]. Pharmacological inhibitors of enzymes in fatty acid biosynthesis were shown to inhibit not only HCMV replication, but also the replication of influenza, an evolutionarily divergent virus [10]. *De novo* fatty acid biosynthesis does not appear to bear the same importance for the replication of HSV-1 as for HCMV (Figure 3D). This is reflected in the lower sensitivity of HSV-1 replication to 5-tetradecyloxy-2-furoic acid (TOFA) (Figure S6) [10], an inhibitor of acetyl-CoA carboxylase, the first committed enzyme of fatty acid biosynthesis.

### Pyrimidine biosynthesis during HSV-1 infection

**Figure 4:**
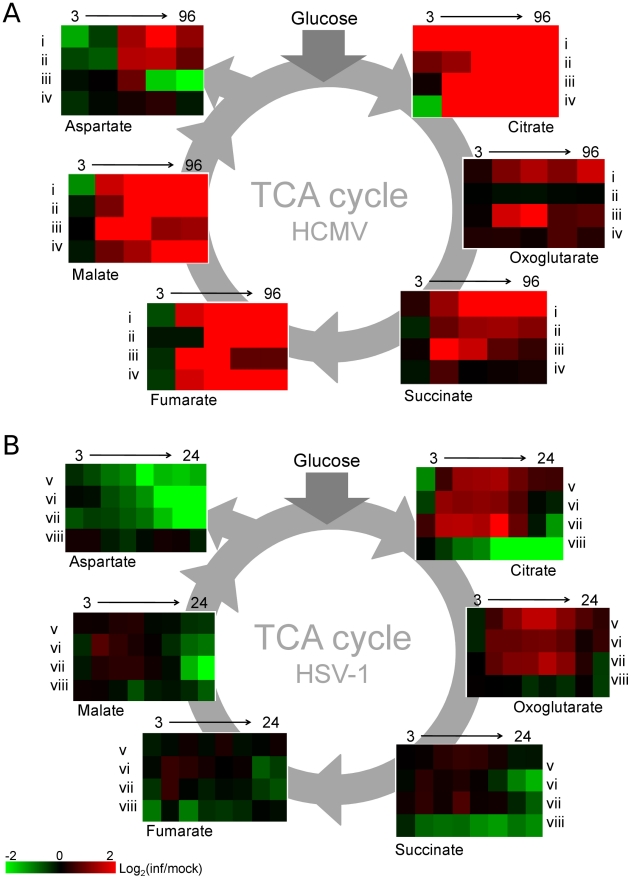
TCA cycle metabolite levels increase in HCMV and drop in HSV-1 infected cells. Plots of individual metabolite abundance during (**A**) HCMV and (**B**) HSV-1 infection. These data are the same as presented in Figure 1. Metabolite concentrations are expressed relative to equivalent mock-treated cells. Rows correspond to infection time courses of the following virus strains and cell types: (i) TB40-HFF, (ii) AD169-HFF, (iii) AD169-MRC5, (iv) TB40-ARPE19, (v) F-HFF, (vi) KOS-HFF, (vii) KOS-MRC5, (viii) KOS-Vero. Columns correspond to time points: 3, 24, 48, 72, 96 hpi for HCMV and 3, 6, 9, 12, 15, 18, 21, 24 hpi for HSV-1.

The reaction catalyzed by pyruvate carboxylase is an anaplerotic reaction that serves to replenish the intermediates of the TCA cycle as they are removed for biosynthetic purposes. However, in spite of its up-regulation during HSV-1 infection, after an initial elevation, the levels of TCA cycle intermediates drop (Figure 4). This indicates that HSV-1 triggers an even greater increase in TCA cycle efflux. Notably, the concentration of aspartate, which is produced from oxaloacetate, decreases significantly after infection with HSV-1. In addition to being used for protein synthesis, aspartate is a substrate for pyrimidine nucleotide biosynthesis.

**Figure 5:**
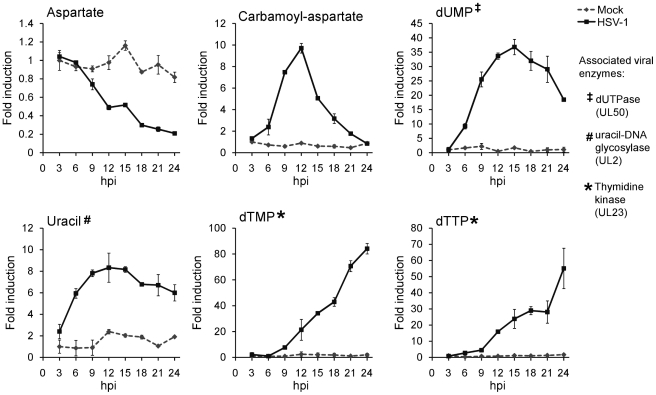
Upregulation of pyrimidine nucleotide biosynthesis during HSV-1 infection. Individual metabolite abundance in HSV-1-infected and mock-treated quiescent HFFs. To show separately the trend in mock versus infected cells, metabolite concentrations are expressed relative to the average level measured in mock-infected cells at 3 h post mock treatment (mean ±2 s.e.; n = 2).

Unlike in HCMV infection, in response to HSV-1 infection the rates of total RNA and total protein syntheses drop [23], [24]. At the same time, viral DNA synthesis increases the demand for deoxyribonucleotides. The nucleotide precursors essential for DNA synthesis can be acquired through salvage reactions or *de novo* synthesis [25], [26]. When replicating in quiescent cells as opposed to actively dividing ones, viruses face a greater challenge in acquiring nucleotides for viral DNA replication, because the *de novo* nucleotide biosynthesis pathways are less active [27]. HSV-1 encodes a set of enzymes addressing this problem and their impact is reflected in increased concentrations of the intermediates of the pyrimidine nucleotide biosynthesis pathway (Figure 5). HCMV employs an alternative mechanism whereby the host cell is driven from quiescence to the G1/S boundary of the cell cycle [28], stimulating host cell nucleotide biosynthesis but preventing host DNA replication. Interestingly, in HSV-1 infected serum-starved fibroblasts dTTP levels are not observed to peak and drop after 6 hpi as reported in Vero cells (Figure 1) [26], and BHK cells [29]. In growth arrested fibroblasts the dTTP pool continues to rise throughout the infection (Figure 5). Such a trend was previously observed in mutant BHK cells that lack thymidine kinase and deoxycytidine kinase activities [29]. Confluent, serum-starved fibroblasts may present a similar cellular environment, with very low basal activity of DNA-biosynthetic enzymes.

Uracil can occur in DNA as a result of cytosine deamination or misincorporation of dUTP [30]. The UL50 and UL2 genes of HSV-1 encode enzymes that address these problems. The viral dUTPase (UL50) serves to reduce incorporation of uracil into viral DNA by decreasing dUTP levels and producing dUMP. Uracil-DNA glycosylase (UL2) participates in base excision repair of the HSV-1 genome, removing uracil from viral DNA [31], [32]. These two viral enzymes are likely responsible for the increased dUMP and uracil levels during HSV-1 infection (Figure 5).

While there is no known HSV-1 gene that causes the increased production of carbamoyl-aspartate, evidence for the regulation of aspartate transcarbamoylase during adenovirus infections has been presented in the past [33], [34]. Furthermore, carbamoyl-aspartate levels are observed to rise dramatically in both HCMV and HSV-1 infections (Figure 1) [10]. Carbamoyl-aspartate is produced by the multifunctional CAD protein, which catalyzes the first three steps of *de novo* pyrimidine biosynthesis in mammalian cells. CAD is highly regulated by growth state-related signaling molecules, such as the epidermal growth factor [35], [36]. Epidermal growth factor receptor has been shown to play a role in the entry of several different viruses, and it or related signaling pathways might contribute to virally-induced increases in carbamoyl-aspartate levels [37], [38], [39].

**Figure 6:**
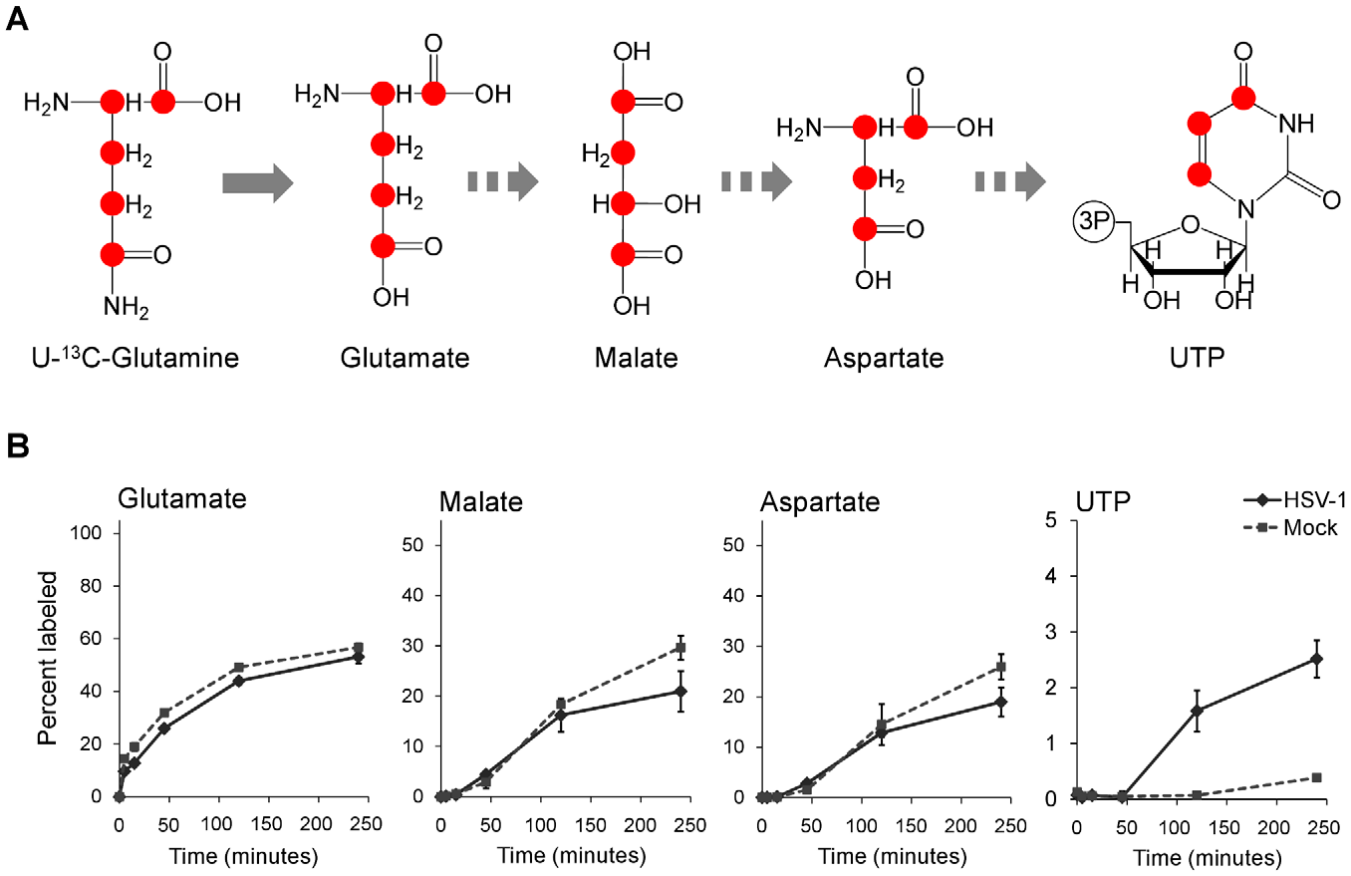
Flux to pyrimidine nucleotide synthesis induced by HSV-1 infection. (**A**) Schematic of carbon labeling from glutamine to UTP arising during carbon influx from glutamine to the TCA cycle. Red dots denote ^13^C atoms originating from uniformly ^13^C-labeled glutamine. (**B**) Plots show the levels of the labeled form of the indicated metabolites expressed as percent of the total metabolite pool. The labeling arose upon switching HSV-KOS infected or mock treated HFF cells to uniformly ^13^C-labeled glutamine media. HCMV infected cells were switched at 48 hpi, HSV-1-infected cells at 12 hpi. The x-axis indicates time post media switch (mean ±2 s.e.; n = 2).

To confirm that flux from aspartate to pyrimidine nucleotides is up-regulated in HSV-1 infection, we analyzed the labeling pattern of the pathway intermediates after switching cells to medium containing uniformly labeled ^13^C-glutamine (Figure 6A). As glutamine contributes to anapleurosis in both mock and infected cells, this resulted in labeling of aspartate in both cases, and thus enabled direct comparison of pyrimidine synthesis between these two conditions. Significantly faster labeling of the pyrimidine end-product UTP was observed in infected cells (Figure 6B). As the concentration of UTP is also elevated in HSV-1 infected cells, flux from aspartate to nucleotide synthesis is markedly increased.

**Figure 7:**
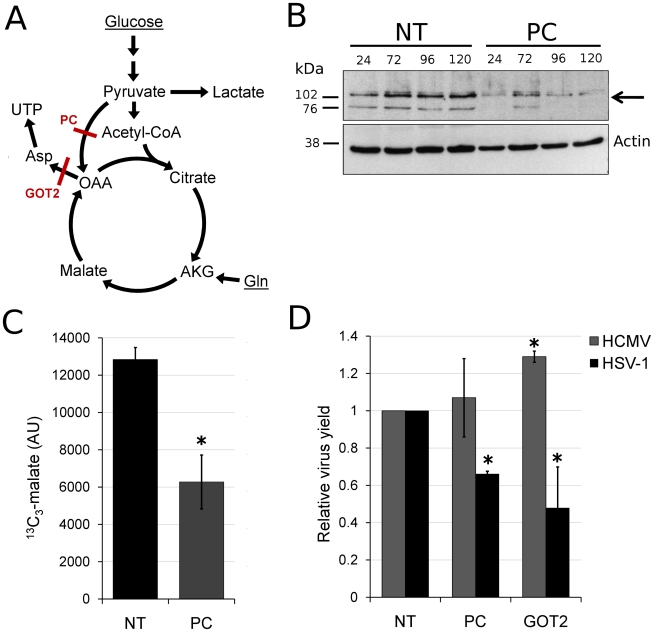
HSV-1 replication is inhibited by reducing flux from glucose toward pyrimidine nucleotide synthesis. (**A**) Schematic diagram of glucose flux to pyrimidine nucleotide biosynthesis. Red lines mark siRNA-targeted reactions catalyzed by pyruvate carboxylase (PC) and aspartate transaminase 2 (GOT2). (OAA: oxaloacetate, AKG: oxoglutarate, Gln: glutamine). (**B**) RNA interference knockdown of pyruvate carboxylase (marked by arrow) in MRC5 cells. Cells were transfected with non-targeting siRNAs (NT) or siRNAs targeting pyruvate carboxylase (PC) and harvested at indicated time points after transfection. Pyruvate carboxylase levels in the cells were detected by western blot using specific antibodies. Beta-actin was employed as a loading control. (**C**) Buildup of ^13^C_3_-labeled malate after switching MRC5 cells to uniformly ^13^C-labeled glucose medium for 2 hours at 10 hpi of HSV-1 (F) infection. The cells have been transfected with a universal non-targeting siRNA (NT) or an siRNA targeting pyruvate carboxylase (PC) 120 h prior to infection (significance: p = 0.007). Symbols indicate experimental data points ±1 s.d.; n = 3; values are given in arbitrary units. (**D**) Production of infectious HSV-1 (F) and HCMV (AD169) virions in cells transfected with siRNAs against pyruvate carboxylase (PC), aspartate transaminase 2 (GOT2), or a universal negative control (NT). The transfection and infection of MRC5 cells were performed as described in Materials and Methods. Values are expressed relative to non-targeting control (±1 s.d.; n = 3). Conditions resulting in significantly altered virus production (p≤0.05) compared to treatment with the universal negative control are marked with a star.

Taken together, the above observations indicate an upregulation of flux in HSV-1 infected cells from glucose to *de novo* pyrimidine nucleotide biosynthesis via the pyruvate carboxylase-catalyzed anaplerotic and the aspartate transaminase 2 catalyzed cataplerotic reactions of the TCA cycle (Figure 7A). In agreement with this, small interfering RNA (siRNA) mediated knockdown of pyruvate carboxylase and aspartate transaminase 2 inhibit HSV-1 replication, but not HCMV (Figure 7B–D).

## Discussion

**Figure 8:**
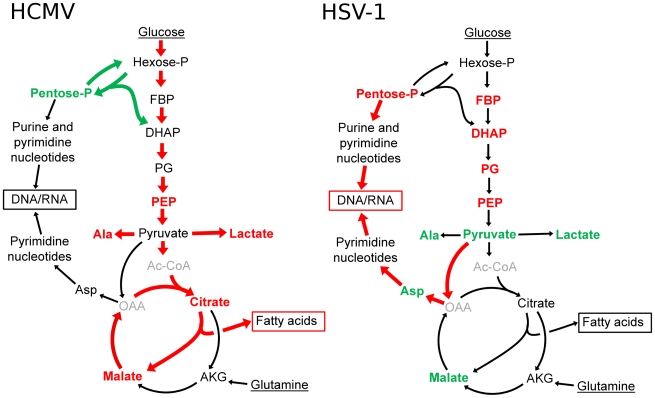
Divergent effects of HCMV and HSV-1 on central carbon metabolism. Schematic summary of major metabolite concentration and flux changes in response to HCMV (left panel) and HSV-1 (right panel) infection of growth arrested fibroblasts. Arrow colors denote flux changes and font colors denote metabolite level changes relative to the mock-treated control (red-increased, green-decreased, grey-not detected). (Hexose-P: glucose-6-phosphate and its isomers, Pentose-P: ribose-phosphate and its isomers, FBP: fructose-1,6-bisphosphate, DHAP: dihydroxy acetone-phosphate, PEP: phosphoenolpyruvate, Asp: aspartate, Ala: alanine, Gln: glutamine, AKG: oxoglutarate, OAA: oxaloacetate, Ac-CoA: acetyl-coenzymeA.)

Viral replication depends on the energy and biosynthetic precursors supplied by host cell metabolism. Using a mass spectrometry-based metabolomic approach we demonstrate that two closely related viruses, HCMV and HSV-1, implement divergent metabolic programs (Figure 1 and Table S1). These programs are robust to host cell type and virus strain. While HCMV enhances glycolytic flux and the delivery of carbon from glucose to the TCA cycle to fuel fatty acid biosynthesis, HSV-1 gears central carbon metabolism toward the production of pyrimidine nucleotide components (Figure 8). The focus of HSV-1, but not HCMV, on nucleotide metabolism is interesting in light of nucleoside analogues (acyclovir and ganciclovir, respectively) being more effective treatments for HSV-1 than for HCMV [40]. Both compounds depend on phosphorylation by viral kinases for their activation, and the metabolic profile of HSV-1 infected cells reflects the activity of the virally encoded thymidine kinase. On the other hand, the only functional HCMV kinase (UL97) is a protein kinase and has little to no nucleotide kinase activity [41]. This difference is reflected in the metabolome and in the lower efficacy of the nucleoside analogues for HCMV. In contrast, we show that TOFA, an inhibitor of the committed step of fatty acid synthesis and elongation, preferentially targets HCMV over HSV-1 (Figure S6).

The viruses also induce robust changes outside of core metabolism. For example, HCMV, but not HSV-1, induces a striking increase in acetylated amino acids (Figure 1). After HSV-1 infection, NAD+ levels dropped by a factor of about 10, but little decline was evident after HCMV infection. We have recently discovered that this NAD+ depletion is due to elevated poly-ADP-ribose polymerase activity (L. Vastag, unpublished work). The activation of poly-ADP-ribose polymerase has also been observed in HIV-1 and Sindbis Virus infected cells [42], [43], [44]. Understanding the significance of such observations requires further study.

Why do these two related viruses induce markedly different changes in host cell metabolism? Both must synthesize viral proteins and nucleic acids and both produce enveloped virions. Perhaps the difference results in part from the markedly different speeds at which the two viruses progress through their replication cycles. HSV-1 produced maximal yields in fibroblasts or epithelial cells within about 24 h, whereas HCMV did not achieve maximal yields until about 96 hpi (Figure S2). One might speculate, then, that HSV-1, which accumulates its DNA fairly rapidly, must elevate nucleotide biosynthesis; in contrast, HCMV, which accumulates its DNA over a much longer time frame, does not require such a strong induction (Figure 1). It is more difficult to suggest why HCMV depends on *de novo* fatty acid biosynthesis more strongly than HSV-1 (Figure S6). It is conceivable that HCMV induces the production of new membranes to serve as a source for the virion envelope, while HSV-1 virions are built from pre-existing membranes. Consistent with this view, HCMV-infected cells develop a well-defined, membranous assembly compartment during the late phase of infection [45], [46], [47], but no equivalent structure has been described within HSV-1-infected cells.

The metabolic program induced by herpes viruses could be implemented in several ways. One potential strategy involves perturbation of general host biochemical milieu. For example, the HCMV UL37x1 protein elevates free intracellular calcium levels [48], which could potentially activate glycolysis through the action of calcium-sensitive kinases [19]. Alternatively, virus-coded gene products could modify or interact with pivotal regulators of host cell metabolism, e.g., the HCMV UL38 protein [49], or with metabolic enzymes themselves to alter their activity. Yet other strategies could involve modulation of host cell enzyme concentrations through mechanisms involving transcription, translation, or protein stability. A comprehensive systems level analysis, incorporating transcriptomic [50], [51], [52], [53], proteomic [8], and metabolic data should help clarify the relative significance of these latter mechanisms.

In addition to elucidating the mechanisms underlying host cell metabolic hijacking, an important priority is defining the metabolic programs of other viruses. Among herpes viruses, it will be interesting to see whether most fit either the HSV-1 or HCMV prototype, or whether alternative programs exist. For smaller viruses, it will be interesting to see whether their yet more precious genome space includes instructions for extensive host cell metabolic reprogramming. Such work holds substantial practical value, given overarching importance of enzyme inhibitors as antivirals and the utility of metabolomics for identifying new antiviral targets.

## Materials and Methods

### Cells and viruses

Primary human foreskin fibroblasts (HFFs) were collected previously [54] and stored in liquid nitrogen. We used them at passages 8–13. ARPE19 human retinal pigment epithelial cells, Vero green monkey kidney epithelial cells and MRC5 human embryonic lung fibroblasts were purchased from the American Type Culture Collection. Cells were grown in Dulbecco's modified Eagle Medium (DMEM) with 10% fetal bovine serum, 100 µg/mL penicillin and streptomycin (Invitrogen), and 4.5 g/L glucose. HSV-1 strain F [55] was kindly provided by B. Roizman (University of Chicago), the HSV-1 KOS 1.1 strain [56] was a gift from D. Hargett (Princeton University), and both viruses were grown in Vero cells [57]. BAD*wt*-GFP is a phenotypically wild-type HCMV laboratory strain that was generated from a bacterial artificial chromosome (BAC) clone of strain AD169 [58] engineered to express green fluorescent protein [59]. TB40/E-eGFP is a phenotypically wild-type HCMV clinical isolate that was derived from a bacterial artificial chromosome termed TB40-BAC4 [60] containing a green fluorescent protein marker gene under control of the SV40 promoter between US34 and TRS1. HCMV strains were grown in MRC-5 cells. To prepare virus stocks for both HSV-1 and HCMV, the media of infected cells was layered over a sorbitol cushion (20% sorbitol, 50 mM Tris-HCl, pH 7.2, 1 mM MgCl_2_) and virus was pelleted by centrifugation (20,000 rpm, 1 h, 4°C, Beckman SW28 rotor). Virus stocks were prepared in DMEM with 0.5% bovine serum albumin and without fetal bovine serum, to avoid serum stimulation of the growth arrested fibroblasts during inoculation.

For analysis of metabolites, fibroblasts (HFFs or MRC5) were grown to confluence and maintained in the presence of serum for 5 d. Cells were then washed with serum-free DMEM and maintained in serum-free DMEM for 24 h before infection or mock treatment. Epithelial cells (ARPE19 or Vero) were grown to 80–90% confluence in DMEM with 10% dialyzed serum (Gemini Bio-Products) before infection. At the time of infection cells were inoculated with virus resuspended in DMEM with or without serum. Mock treated cells were inoculated with equivalent, virus-free DMEM. After a 1 h inoculation fresh DMEM was added to the cells, following two washes with the appropriate medium. For each time point in every experiment an additional mock treated and infected plate was processed for packed cell volume measurement. Approximately 5×10^5^ cells were added to packed cell volume tubes (Techno Plastic Products), which were centrifuged at 2000×g for 5 min before reading [61]. Packed cell volume measurements were used to normalize the metabolite levels between samples.

### Metabolite extraction

At various times post infection or addition of ^13^C-labeled glucose- or glutamine-containing DMEM, the media of infected and mock cells was aspirated and −80°C, 80∶20 methanol∶water (v/v) was immediately added to quench metabolism. There were no washing steps prior to metabolism quenching, as such steps risk metabolic alterations. Metabolites were then extracted as described previously [21]. The extract was dried under nitrogen and metabolites were resuspended in HPLC-grade water and centrifuged at 15000×g speed for 5 min to remove particulate matter before analysis. To minimize complications due to excessive sample concentration and associated ion suppression during LC-MS analysis, samples were diluted substantially prior to analysis: metabolites collected from 10^6^ cells (a confluent 60 mm plate of fibroblasts) were resuspended in 500 µL water.

### Liquid chromatography – mass spectrometry

To quantitatively measure the levels of metabolites in extracts prepared from infected or mock treated cultured mammalian cells, two different mass spectrometry methods were employed. Liquid chromatography-tandem mass spectrometry (LC-MS/MS) in selective reaction monitoring (SRM) mode was used to assay for ∼200 metabolites of confirmed identity from a wide range of metabolic pathways [62]. A Finnigan TWQ Quantum Ultra mass spectrometer was used in the positive ionization mode, and a TSQ Quantum Discovery MAX mass spectrometer in the negative mode, each equipped with an electrospray ionization source (Thermo Fisher Scientific). The SRMs were constructed with parameters acquired through optimizing the collision induced fragmentation of purified standards of the given metabolites. The LC method in positive mode employed an aminopropyl column for separation [62], while in negative mode the metabolite extracts were passed through a C18 column using tributylamine as an ion pairing agent to achieve longer retention of polar compounds [63], [64]. In addition, the LC-MS/MS method was complemented with untargeted analysis using liquid chromatography coupled to a stand-alone orbitrap mass spectrometer (Thermo Fisher Scientific Exactive instrument) which performs full scans from 85 to 1000 m/z at 100,000 mass resolution [65]. In this system, identification of compounds is based on two parameters: the retention time on the LC column and the compound mass measured with less than 2 ppm mass accuracy. Peaks were identified and peak heights exported with the Metabolomic Analysis and Visualization Engine (MAVEN) [66].

### Glucose uptake and lactate excretion

For glucose uptake and lactate excretion measurements, media samples were collected every 3 h between 45 and 57 hpi for HCMV, and every 2 h between 6 and 18 hpi for HSV-1. The concentrations of lactate and glucose were measured using a YSI 7100 Select Biochemistry Analyzer (YSI Incorporated). Uptake and excretion rates were determined as the rate of concentration change of these compounds in the media. The values were corrected using the packed cell volume of the infected and mock cells.

### Metabolic flux analysis

For experiments involving monitoring the rate of incorporation of ^13^C-labeled nutrient into downstream metabolites, cells were switched to fresh media 1 h before addition of the labeled nutrient. This minimized the perturbation to the cells when their medium was replaced with isotope containing medium. Cells were then maintained in medium containing the labeled nutrient for different lengths of time. Metabolites were extracted and various isotopically labeled forms quantified by mass spectrometry. The values were corrected for the natural abundance of ^13^C as described previously [10]. Labeled DMEM was prepared from glucose and glutamine-free DMEM with the addition of U-^13^C-glucose or U-^13^C-glutamine (Cambridge Isotope Laboratories). All media were equilibrated to the incubator temperature and gas composition before use.

### siRNA transfection

Double stranded siRNA molecules directed against pyruvate carboxylase (5′-GACUGUACGCGGCCUUCGATT), aspartate transaminase 2 (5′-CUAUUGAGAGCUUCACACATT), and a Universal Negative Control (SIC001) were purchased from Sigma. Subconfluent MRC5 cells seeded into 96-well plates were transfected with 10 pmol of siRNA using Oligofectamine transfection reagent (Invitrogen) according to the manufacturer's instructions. For HCMV experiments, the siRNA transfected cells were incubated for 24 hours and then infected with HCMV strain BAD*wt*-GFP at a multiplicity of 0.1 pfu/cell. The cells were further incubated for 96 hours and media containing the infectious virus were harvested. Since HSV-1 replicates with a faster kinetics than HCMV, the transfected cells were incubated for 3 days to allow efficient knockdown of target gene. The cells were then infected with HSV-1 strain F at a multiplicity of 0.02 pfu/cell and media were harvested 24 hours after infection. The yield of HCMV and HSV in the media was determined by infectious focus assay. Briefly, fresh MRC5 cells were infected with different dilutions of viruses and fixed 24 hours after HCMV or 4 hours after HSV-1 infection with methanol at −20°C. Foci were identified using mouse monoclonal primary antibodies to HCMV immediate early IE1 protein (1B12) [54] or HSV-1 immediate early ICP4 protein [67] and a goat anti-mouse Alexa Fluor 488-conjugated secondary antibody (Invitrogen).

### Western blot analysis

MRC5 cells were seeded into 6-well dishes and transfected at 70% confluence as described above. HFF cells were grown to confluence, serum starved for 24 hours and infected at 3 pfu/cell with HSV-1 (F strain). At selected times post transfection of MRC5 cells and infection of HFFs, cells were washed with phosphate-buffered saline (PBS), harvested and stored at −80°C. Cells were lysed in RIPA-light buffer (50 mM Tris-HCl, pH 8.0, 1% NP-40, 0.1% SDS, 150 mM NaCl, 0.1% Triton X-100, 5 mM EDTA) with protease inhibitors (Roche Applied Science), and protein concentrations were determined by Bradford assay. Proteins were separated by 10% SDS-containing polyacrylamide gel electrophoresis and transferred to nitrocellulose membranes. Membranes were probed with a primary rabbit polyclonal antibody directed against pyruvate carboxylase (NBP1-49536, Novus) at a dilution of 1∶1000 in PBS-T and 1% nonfat milk. After washing with PBS-T, membranes were probed with goat anti-rabbit HRP-coupled secondary antibodies diluted 1∶5000 in PBS-T containing 1% milk. Proteins were visualized by chemiluminescence using the ECL detection system (Amersham).

### Tests of statistical significance

All p-values were calculated by two-tailed, non-paired T-test.

### Accession numbers

Pyruvate carboxylase (PC): P11498, aspartate transaminase 2 (GOT2): P00505, carbamoyl-phosphate synthetase 2, aspartate transcarbamylase, and dihydroorotase (CAD): P27708, HSV-1 dUTPase (UL50): P10234, HSV-1 uracil-DNA glycosylase (UL2): P10186, HSV-1 thymidine kinase (UL23): P03176.

## Supporting Information

Figure S1 **Steady state metabolite levels measured in MRC5, HFF, ARPE19 and Vero cells.** The heatmaps show levels of metabolites measured in biological replicates for each cell type (numbered), normalized by packed cell volume and expressed relative to the average level of the particular metabolite across all cell types. Ratios are log transformed and plotted on a color scale. Metabolites in panel (**A**) are clustered by uncentered Pearson correlation, and in panel (**B**) they are presented in the same order as in Figure 1. HFFs and MRC5s were confluent for 4 d and serum starved for 24 h prior to analysis. ARPE19 and Vero cells were 80% confluent and actively replicating in the presence of dialyzed serum at the time of extraction. Rows correspond to metabolites measured either by LC-high resolution MS or LC-triple quadrupole MS/MS (those measured by triple quadruople are marked “QQQ”). Columns correspond to biological replicates.
(TIF)

Figure S2 **One-step viral growth curves of HCMV and HSV-1.** Supernatants of cells, which were extracted for metabolomic analysis, were collected and the titered by TCID_50_ limiting dilution assay. Virus titers at various hours post infection are plotted on a log scale (mean ±1 s.d.; n = 2).
(TIF)

Figure S3 **Divergent metabolic profiles of HCMV and HSV-1 infected cells.** This figure is a replicate of Figure 1 of the main text, except it is presented using a yellow-blue color scale for readers with difficulty distinguishing red-green hues.
(TIF)

Figure S4 **Singular value decomposition of the metabolome matrix shown in** **Figure 1** **.** (**A**) Vectors were ranked based on the percent of information they accounted for, and the top 25 vectors were plotted on a color scale. The first three rows correspond to the vectors plotted in Figure S4B. (**B**) The three most significant characteristic vectors. The signal of each characteristic vector is plotted versus time. The vectors include entries for each time point during the eight infection time courses. Time courses are arranged in the same order as in Figure 1. Cell types, virus strains and increasing hpi are indicated on the figure. Time points were 3, 24, 48, 72, 96 hpi for HCMV, with also a 120 hpi sample for the infection of ARPE19 cells, and 3, 6, 9, 12, 15, 18, 21 and 24 hpi for HSV-1. The first vector (generic response to infection) accounts for 16% of the information from the dataset, while the second (virus specific response) captures 12%. The third vector accounts for 6% of the information in the matrix, and highlights the differential metabolic response to HSV-1 in Vero cells (the last segment of the eight).
(TIF)

Figure S5 **HSV-1 induced anapleurotic flux into malate.** Levels of various labeled forms of malate expressed as percent of the total malate pool upon switching HSV-1 KOS-infected or mock-treated HFF cells to uniformly ^13^C-labeled glucose medium at 12 hpi. The x-axis indicates time after switching to labeled media (mean ±1 s.d.; n = 2).
(TIF)

Figure S6 **Effect of inhibiting acetyl-CoA carboxylase on HCMV and HSV-1 replication.** (**A**) Production of infectious HCMV (AD169) and HSV-1 (KOS) virions in the presence of carrier (DMSO) or the indicated concentrations of the acetyl-CoA carboxylase inhibitor TOFA in confluent, serum-starved HFF cells. Supernatants of cells infected at a multiplicity of infection of three were collected at 96 hpi from HCMV and 24 hpi from HSV-1 infected cells, and titered by TCID_50_ limiting dilution assay. Virus titers at various hours post infection are plotted on a log scale (mean ±1 s.e.; n (HSV-1) = 4, n (HCMV) = 3). (**B**) Cell viability of HSV-1 infected cells based on trypan blue exclusion at 24 hpi in the presence or absence of TOFA. Trypan blue stain was added to an aliquot of cells to assess live/dead ratio. For each condition at least 500 cells were counted.
(TIF)

Table S1 **Metabolite levels during the course of HCMV and HSV-1 infection.** Values are normalized by packed cell volume and expressed relative to levels measured in the equivalent mock treated host cells. Rows correspond to metabolites measured either by LC-high resolution MS or LC-triple quadrupole MS/MS (those measured by triple quadruople are marked “QQQ”). Columns correspond to hours post infection for each of the eight infection time courses. The host cells and virus strains used in each time course are indicated. Values are averages of duplicate independent biological experiments.
(XLS)
