# Supplementary figures and images for: Divergent Effects of Human Cytomegalovirus and Herpes Simplex Virus-1 on Cellular Metabolism
Source: PLoS Pathog. 2011 Jul 14;7(7):e1002124. doi: 10.1371/journal.ppat.1002124 (PMC3136460; doi:10.1371/journal.ppat.1002124)

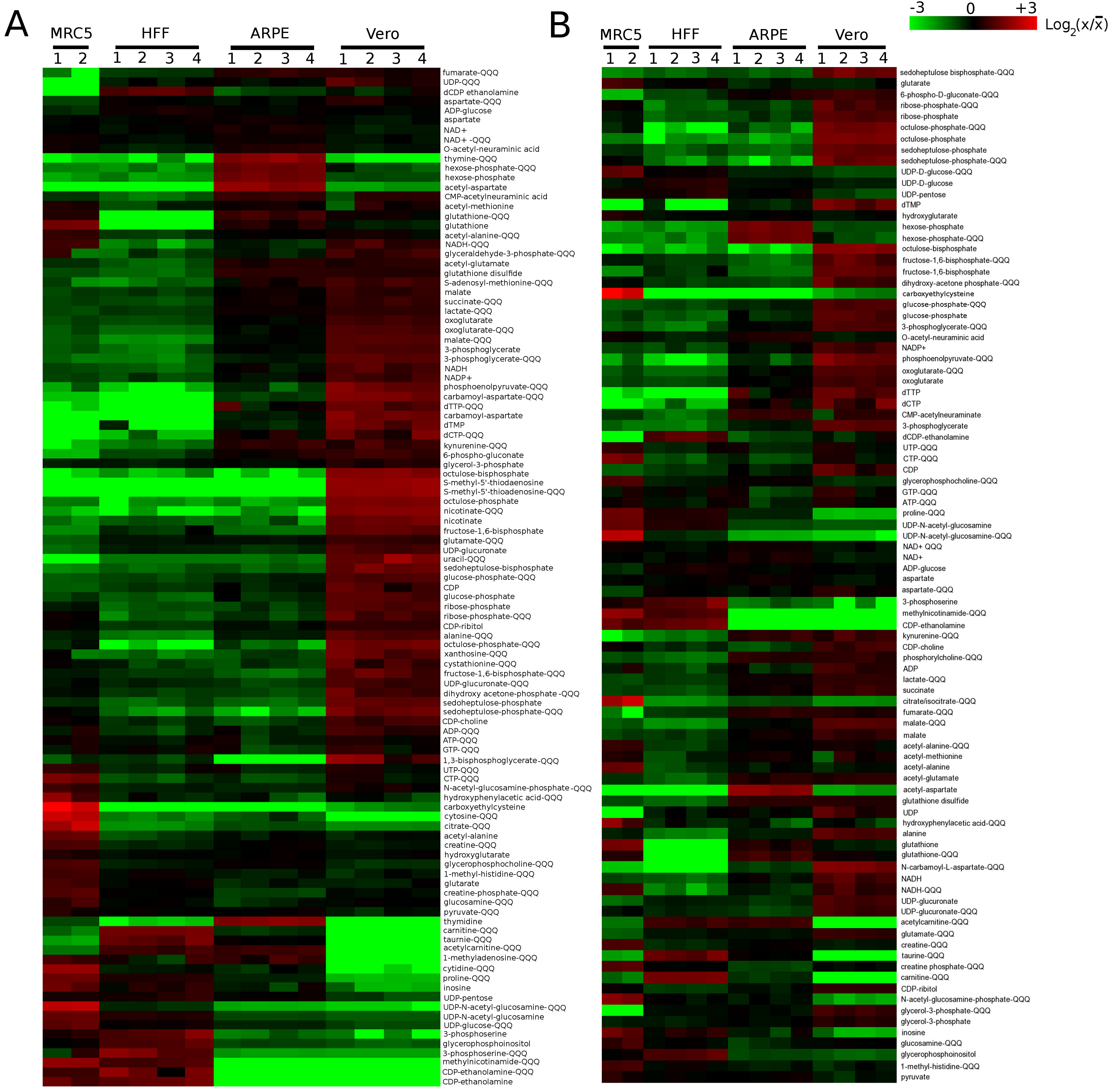

Supplement: Figure S1 — Steady state metabolite levels measured in MRC5, HFF, ARPE19 and Vero cells. The heatmaps show levels of metabolites measured in biological replicates for each cell type (numbered), normalized by packed cell volume and expressed relative to the average level of the particular metabolite across all cell types. Ratios are log transformed and plotted on a color scale. Metabolites in panel (A) are clustered by uncentered Pearson correlation, and in panel (B) they are presented in the same order as in Figure 1. HFFs and MRC5s were confluent for 4 d and serum starved for 24 h prior to analysis. ARPE19 and Vero cells were 80% confluent and actively replicating in the presence of dialyzed serum at the time of extraction. Rows correspond to metabolites measured either by LC-high resolution MS or LC-triple quadrupole MS/MS (those measured by triple quadruople are marked “QQQ”). Columns correspond to biological replicates. (TIF) [file ppat.1002124.s001.tif]

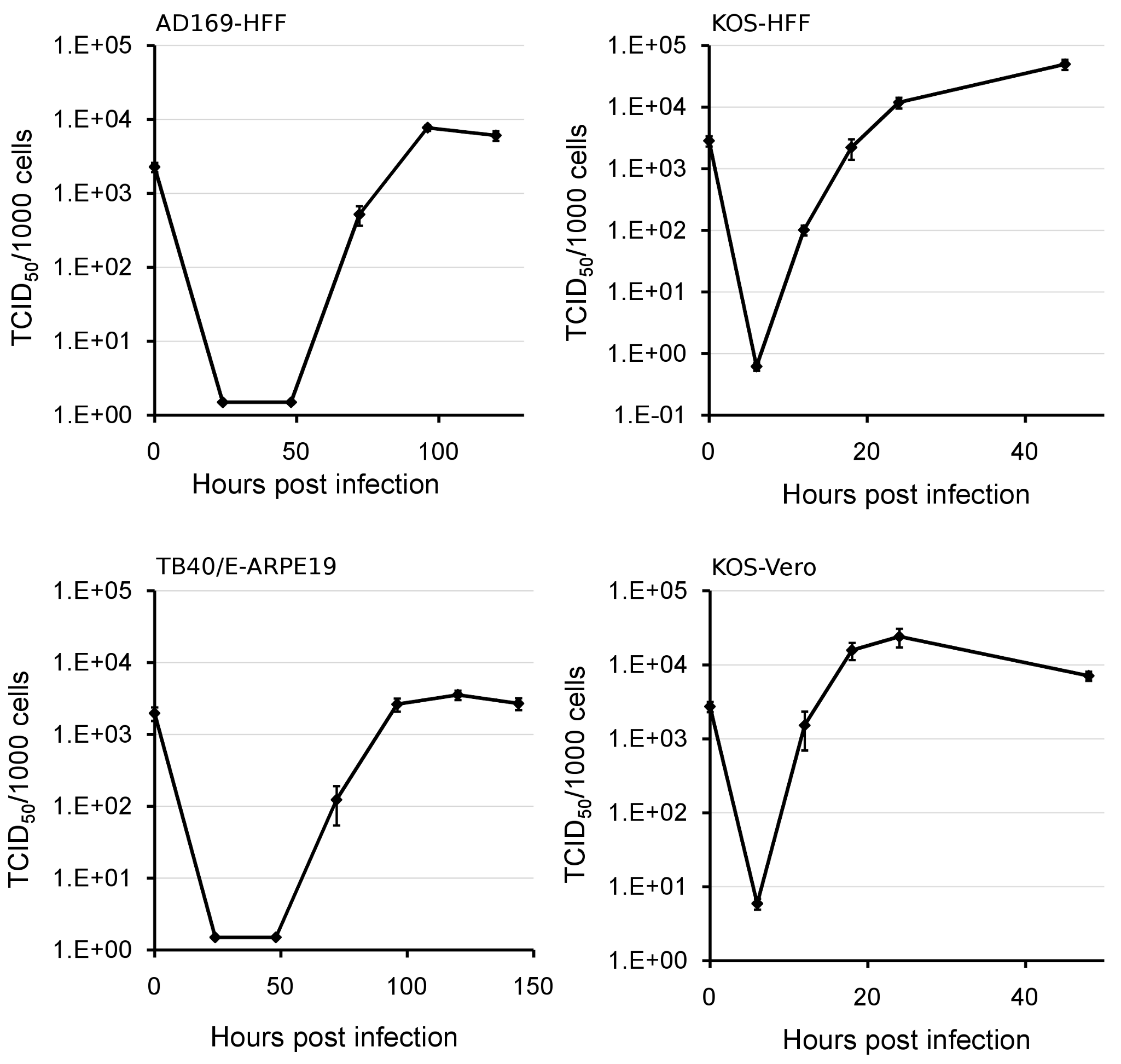

Supplement: Figure S2 — One-step viral growth curves of HCMV and HSV-1. Supernatants of cells, which were extracted for metabolomic analysis, were collected and the titered by TCID50 limiting dilution assay. Virus titers at various hours post infection are plotted on a log scale (mean ±1 s.d.; n = 2). (TIF) [file ppat.1002124.s002.tif]

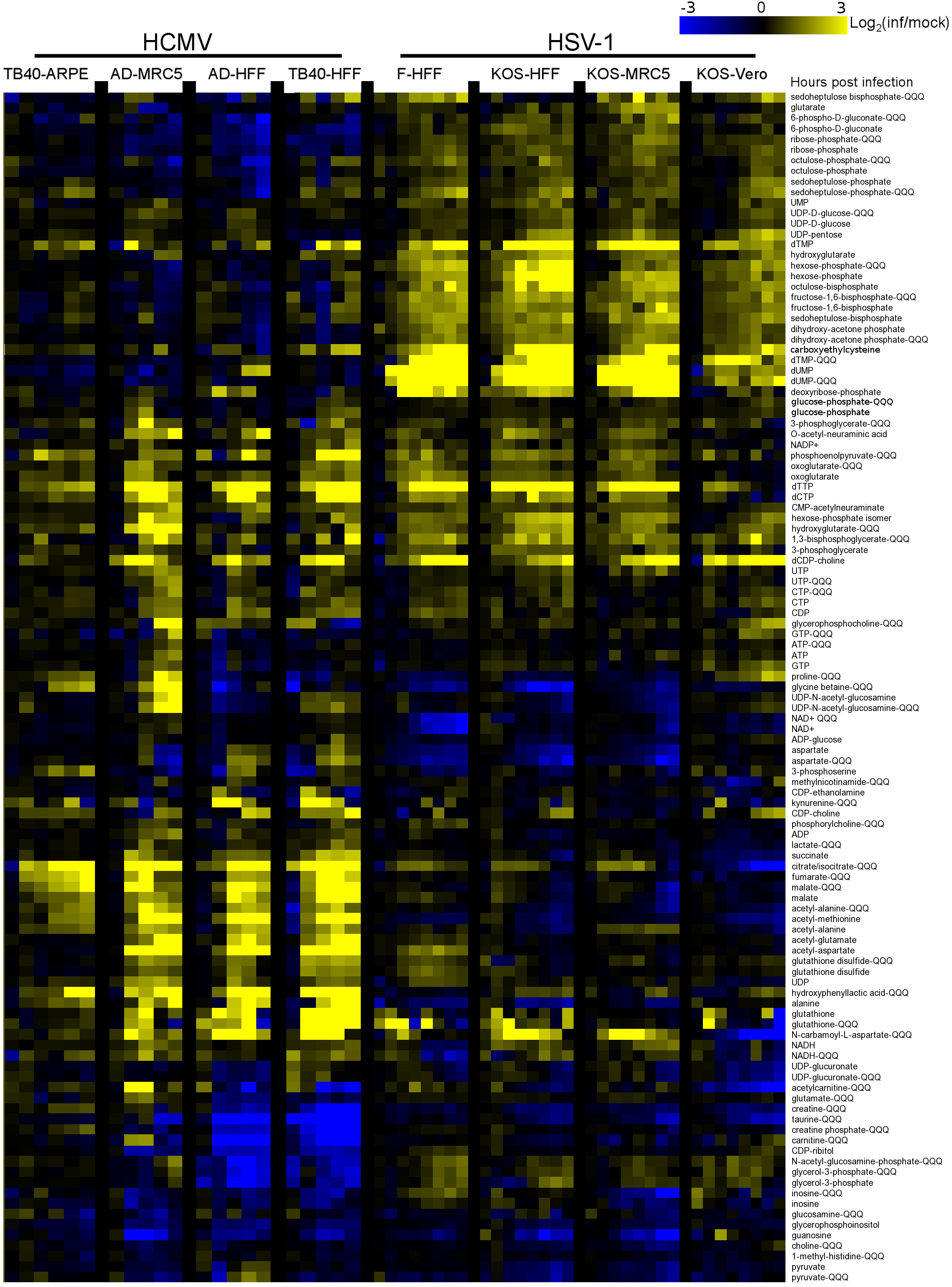

Supplement: Figure S3 — Divergent metabolic profiles of HCMV and HSV-1 infected cells. This figure is a replicate of Figure 1 of the main text, except it is presented using a yellow-blue color scale for readers with difficulty distinguishing red-green hues. (TIF) [file ppat.1002124.s003.tif]

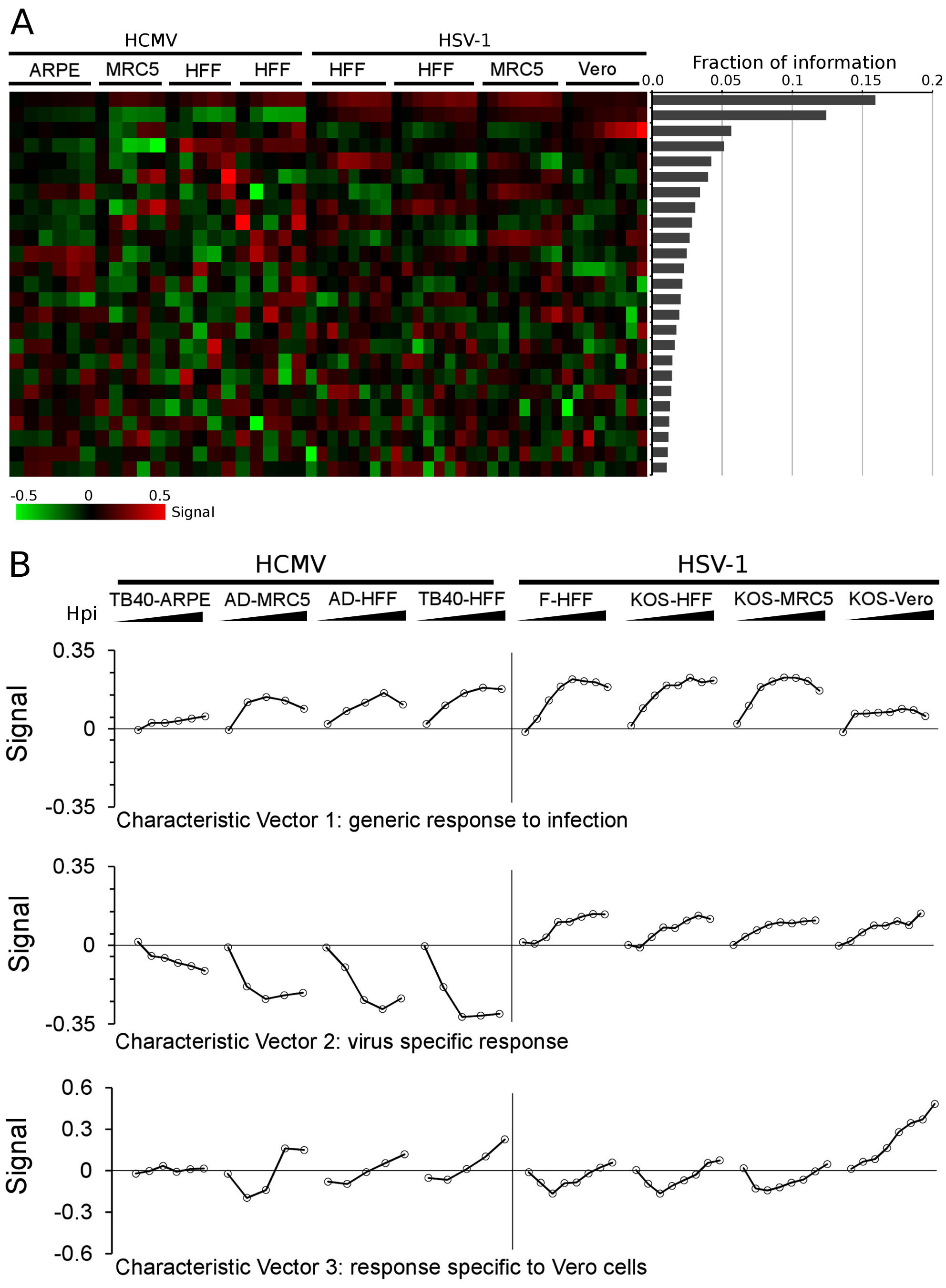

Supplement: Figure S4 — Singular value decomposition of the metabolome matrix shown in Figure 1 . (A) Vectors were ranked based on the percent of information they accounted for, and the top 25 vectors were plotted on a color scale. The first three rows correspond to the vectors plotted in Figure S4B. (B) The three most significant characteristic vectors. The signal of each characteristic vector is plotted versus time. The vectors include entries for each time point during the eight infection time courses. Time courses are arranged in the same order as in Figure 1. Cell types, virus strains and increasing hpi are indicated on the figure. Time points were 3, 24, 48, 72, 96 hpi for HCMV, with also a 120 hpi sample for the infection of ARPE19 cells, and 3, 6, 9, 12, 15, 18, 21 and 24 hpi for HSV-1. The first vector (generic response to infection) accounts for 16% of the information from the dataset, while the second (virus specific response) captures 12%. The third vector accounts for 6% of the information in the matrix, and highlights the differential metabolic response to HSV-1 in Vero cells (the last segment of the eight). (TIF) [file ppat.1002124.s004.tif]

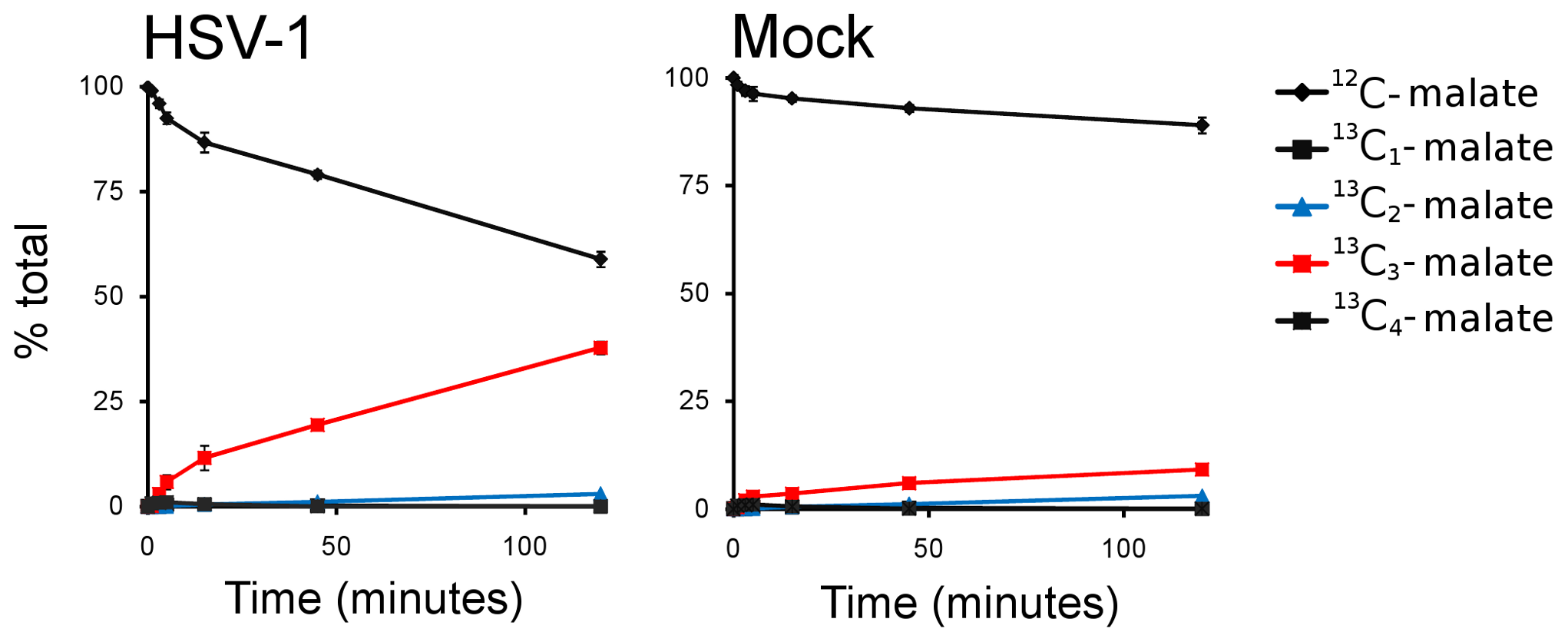

Supplement: Figure S5 — HSV-1 induced anapleurotic flux into malate. Levels of various labeled forms of malate expressed as percent of the total malate pool upon switching HSV-1 KOS-infected or mock-treated HFF cells to uniformly 13C-labeled glucose medium at 12 hpi. The x-axis indicates time after switching to labeled media (mean ±1 s.d.; n = 2). (TIF) [file ppat.1002124.s005.tif]

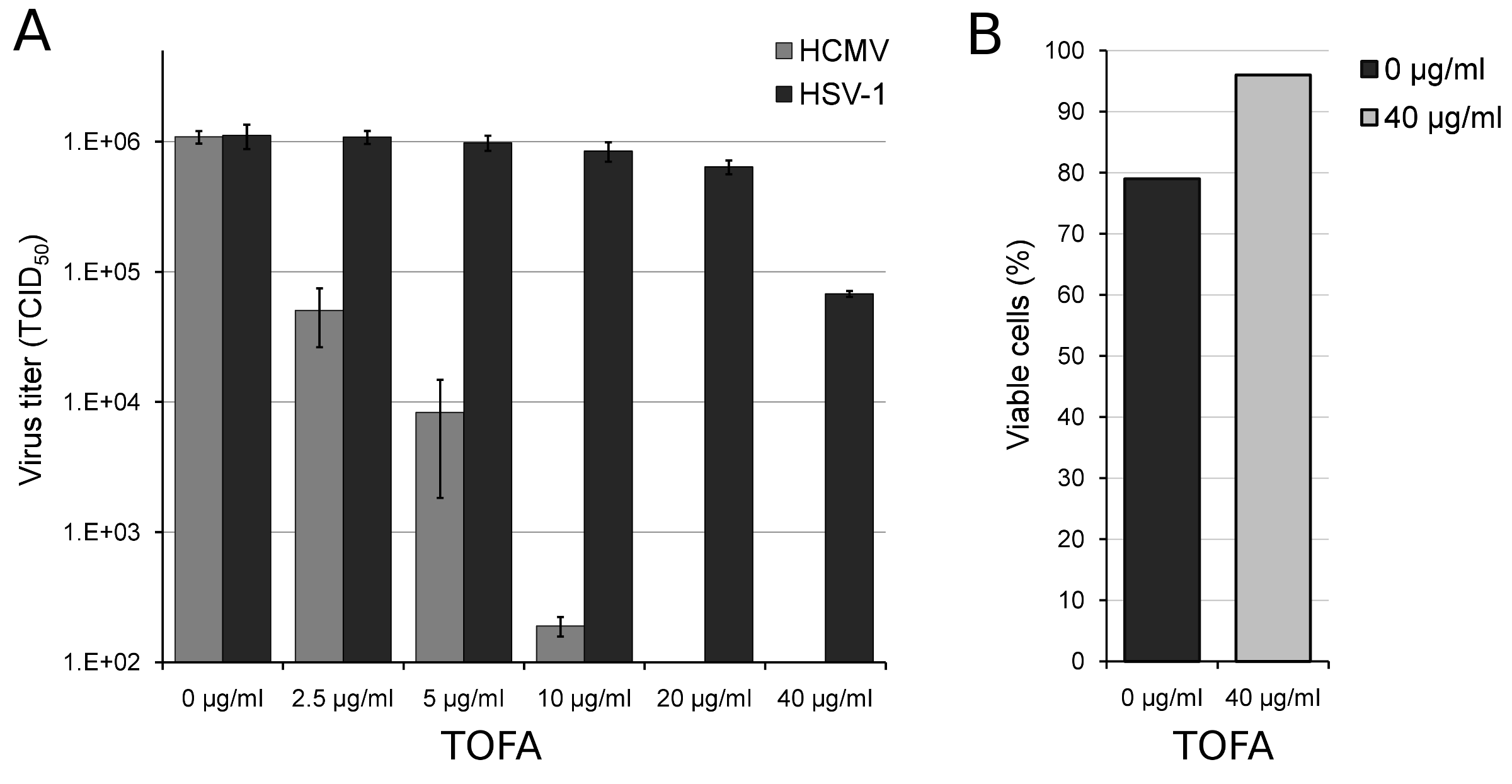

Supplement: Figure S6 — Effect of inhibiting acetyl-CoA carboxylase on HCMV and HSV-1 replication. (A) Production of infectious HCMV (AD169) and HSV-1 (KOS) virions in the presence of carrier (DMSO) or the indicated concentrations of the acetyl-CoA carboxylase inhibitor TOFA in confluent, serum-starved HFF cells. Supernatants of cells infected at a multiplicity of infection of three were collected at 96 hpi from HCMV and 24 hpi from HSV-1 infected cells, and titered by TCID50 limiting dilution assay. Virus titers at various hours post infection are plotted on a log scale (mean ±1 s.e.; n (HSV-1) = 4, n (HCMV) = 3). (B) Cell viability of HSV-1 infected cells based on trypan blue exclusion at 24 hpi in the presence or absence of TOFA. Trypan blue stain was added to an aliquot of cells to assess live/dead ratio. For each condition at least 500 cells were counted. (TIF) [file ppat.1002124.s006.tif]
